# Evidence for peri-lacunar remodeling and altered osteocyte lacuno-canalicular network in mouse models of myeloma-induced bone disease

**DOI:** 10.1093/jbmrpl/ziae093

**Published:** 2024-07-12

**Authors:** Holly Evans, Rebecca Andrews, Fatma Ali Abedi, Alexandria Sprules, Jacob Trend, Goran Lovric, Alanna Green, Andrew Chantry, Claire Clarkin, Janet Brown, Michelle Lawson

**Affiliations:** Division of Clinical Medicine and Mellanby Centre for Musculoskeletal Research, School of Medicine and Population Health, Faculty of Health, University of Sheffield, Sheffield S10 2RX, United Kingdom; Division of Clinical Medicine and Mellanby Centre for Musculoskeletal Research, School of Medicine and Population Health, Faculty of Health, University of Sheffield, Sheffield S10 2RX, United Kingdom; Department of Haematology, Sheffield Teaching Hospitals, Royal Hallamshire Hospital NHS Foundation Trust, Sheffield S10 2JF, United Kingdom; Division of Clinical Medicine and Mellanby Centre for Musculoskeletal Research, School of Medicine and Population Health, Faculty of Health, University of Sheffield, Sheffield S10 2RX, United Kingdom; Division of Clinical Medicine and Mellanby Centre for Musculoskeletal Research, School of Medicine and Population Health, Faculty of Health, University of Sheffield, Sheffield S10 2RX, United Kingdom; School of Biological Sciences, University of Southampton, Southampton SO17 1BJ, United Kingdom; TOMCAT beamline, Swiss Light Source, Paul Scherrer Institut, Forschungsstrasse 111, 5232 Villigen, Switzerland; Division of Clinical Medicine and Mellanby Centre for Musculoskeletal Research, School of Medicine and Population Health, Faculty of Health, University of Sheffield, Sheffield S10 2RX, United Kingdom; Division of Clinical Medicine and Mellanby Centre for Musculoskeletal Research, School of Medicine and Population Health, Faculty of Health, University of Sheffield, Sheffield S10 2RX, United Kingdom; Department of Haematology, Sheffield Teaching Hospitals, Royal Hallamshire Hospital NHS Foundation Trust, Sheffield S10 2JF, United Kingdom; School of Biological Sciences, University of Southampton, Southampton SO17 1BJ, United Kingdom; Division of Clinical Medicine and Mellanby Centre for Musculoskeletal Research, School of Medicine and Population Health, Faculty of Health, University of Sheffield, Sheffield S10 2RX, United Kingdom; Department of Haematology, Sheffield Teaching Hospitals, Royal Hallamshire Hospital NHS Foundation Trust, Sheffield S10 2JF, United Kingdom; Division of Clinical Medicine and Mellanby Centre for Musculoskeletal Research, School of Medicine and Population Health, Faculty of Health, University of Sheffield, Sheffield S10 2RX, United Kingdom

**Keywords:** myeloma, osteocytes, bone, lacuno-canalicular network, synchrotron, peri-lacunar remodeling

## Abstract

Myeloma bone disease (MBD) affects ~90% of multiple myeloma patients, but current treatment options are suboptimal. Therefore, to successfully develop new therapies or optimize current ones, we must improve our fundamental knowledge of how myeloma affects bone microstructure and function. Here, we have investigated the osteocyte lacuno-canalicular network (LCN) in MBD, as bone porosity affects bone quality and resilience. We used the syngeneic 5TGM1-C57BL-Kalwrij and the xenograft U266-NSG models at end stage and compared them to healthy controls (naïve). Micro-computed tomography (μCT) and histomorphometry indicated the 5TGM1 and U266 models developed mild and extensive MBD, respectively, with the U266 model producing large osteolytic lesions. High-resolution synchrotron micro-CT (SR-μCT) revealed significant osteocyte lacunae changes in U266 bones but not 5TGM1, with a reduction in lacunae number and sphericity, and an increase in lacunae volume compared with naïve. Canalicular length, visualized using histological Ploton silver staining, appeared significantly shorter in 5TGM1 and U266 bones compared with naïve. Canalicular area as a proportion of the bone was also decreased by 24.2% in the U266 model. We observed significant upregulation of genes implicated in peri-lacunar remodeling (PLR), but immunohistochemistry confirmed that the osteocyte-specific protein sclerostin, a known driver of PLR, was unchanged between MBD and naïve bones. In summary, we have demonstrated evidence of PLR and altered organization of the osteocyte LCN in MBD mouse models. The next step would be to further understand the drivers and implications of PLR in MBD, and whether treatments to manipulate PLR and the LCN may improve patient outcomes.

## Graphical Abstract

**Graphical Abstract:**
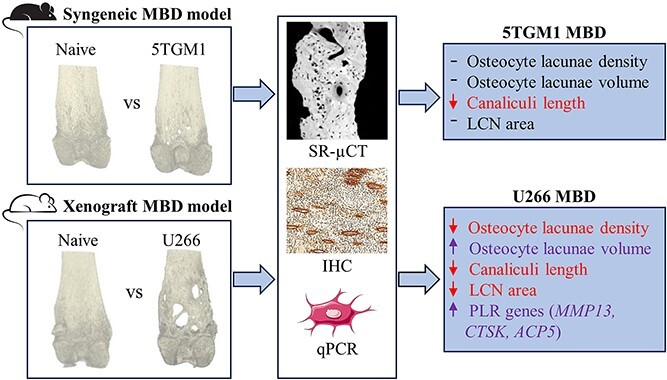

## Introduction

Multiple myeloma is a blood cancer that develops in plasma cells in the bone marrow. Patients often present with myeloma bone disease (MBD) caused by the uncoupling of bone remodeling, resulting in osteolytic lesions and trabecular thinning.^1^ Current therapies, such as bisphosphonates and denosumab (receptor activator of nuclear factor kappa-β ligand (RANKL) inhibitor), target osteoclasts but often leave patients with high fracture risk, possibly due to enhancement and propagation of microcracks over time.^2^ Therefore, to successfully develop new therapies or optimize current ones, we must improve our fundamental knowledge of how myeloma affects bone. Most research to date has focused on osteoblasts and osteoclasts, with limited knowledge about osteocytes. Therefore, further research into the role of osteocytes and their lacuno-canalicular network (LCN) in MBD is needed, both to fully understand their part in MBD-induced bone fragility, and to further explore whether osteocytes could be targeted therapeutically to treat MBD.

Osteocytes, comprising >90% of the total bone cell population,^3^ are derived from terminally differentiated cells of the osteoblast lineage. They reside in lacunae embedded within the mineralized bone matrix and form a dense, intricate dendritic network that allows them to communicate with each other and other bone cells via the LCN.^4^ Osteocytes are key regulators of bone remodeling, coordinating osteoblast and osteoclast proliferation and differentiation through signaling pathways.^5^^,^^6^ They produce sclerostin, a protein encoded by the *Sost* gene that binds to LRP5/6 receptors and inhibits the Wnt pathway, leading to reduced bone formation. Osteocytes also directly control bone homeostasis through a process called peri-lacunar remodeling (PLR), whereby they resorb and replace the extracellular matrix, comprising type I collagen, that directly surrounds them.^7^^,^^8^ PLR has been demonstrated in several mouse lactation studies^9^^,^^10^ but can occur in both male and female mice.^11^ Moreover, it is initiated in conditions such as osteomalacia^12^ and rickets.^13^ Interestingly, sclerostin and TGFβ are known drivers of PLR,^8^^,^^14^ both of which are known to be regulatory factors in myeloma.

Studies have shown that patients with MBD have proportionally fewer viable osteocytes than healthy controls.^15^ In preclinical MBD models, Ziouti et al.^16^ have observed enlarged osteocyte lacunae and disorganization in the osteocyte LCN, but these changes were not quantified. There is mixed data regarding sclerostin expression in MBD. Giuliani et al.^15^ found that sclerostin expression by osteocytes in myeloma patients was not significantly different than that in healthy patients; in contrast, Terpos et al.^17^ reported that patients with symptomatic myeloma had elevated circulating sclerostin compared with healthy patients or those with monoclonal gammopathy of undetermined significance. These differences in sclerostin may be explained by Delgado-Calle et al. who observed that myeloma cells increase osteocyte apoptosis, both in vitro and in vivo, and that myeloma cells upregulate *Sost* (sclerostin gene) mRNA levels in osteocytes in vitro.^18^ Contrastingly, McDonald et al. found minimal difference in *Sost* expression in osteocytes from MBD mice at disease end stage and no difference in the number of sclerostin-positive osteocytes.^19^ Sclerostin expression may, therefore, be dependent on the stage of MBD, limiting efficacy of anti-sclerostin therapies in some patients. However, in preclinical models of MBD, sclerostin inhibition has shown great promise,^19^ but as yet therapies such as romosozumab have not been used clinically in myeloma patients, only in osteoporosis.^20^^,^^21^

Osteocytes play a clear role in bone homeostasis, yet much is unknown about how the osteocyte LCN is implicated in MBD pathogenesis. Here, we hypothesized that MBD leads to increased osteocyte apoptosis, resulting in a reduced and disorganized LCN that we can correlate to sclerostin expression. To test this, we used two mouse models, one with mild MBD (5TGM1) and one with extensive MBD (U266), and then compared their bones to those from healthy control (naïve, non-tumor controls) mice. Using Ploton silver staining and high-resolution SR-μCT, we determined that osteocyte canaliculi appeared shorter in both models, and in the highly osteolytic U266 bones, there were fewer osteocyte lacunae. However, these remaining lacunae were enlarged and the LCN appeared to have an altered organization with reduced area coverage. Isolation of osteocyte-enriched RNA from marrow-flushed U266 whole bones showed that key PLR-related genes were upregulated, implying that PLR may be driving the changes seen in the osteocyte lacunae and the LCN.

## Material and methods

### Ethical approval

All animal experiments were approved by the University of Sheffield Animal Ethics Committee and the UK Home Office (PPL PP3267943) in strict compliance with the Animal (Scientific Procedures) Act 1986.

### Cell lines

5TGM1-GFP and U266-GFP-Luc cells were maintained in RPMI 1640 medium with 10% fetal bovine serum and 50 units/ml penicillin/100 μg/ml streptomycin in an atmosphere of 5% CO_2_ at 37 °C. Cells were confirmed as negative for mycoplasma in the week preceding inoculation.

### In vivo studies

5TGM1 model (syngeneic): 6–8 wk old male C57BL/KaLwRijHsd (BKAL) mice (Charles River Laboratories, UK) were injected intravenously (i.v.) with 2 × 10^6^ 5TGM1-eGFP cells (5TGM1 tumor group, *n* = 8) or PBS (BKAL, naïve control group, *n* = 5). All mice were sacrificed at 3 wk posttumor cell injection.

U266 model (xenograft): 9–10 wk old female NOD *scid* gamma (NOD.Cg-*Prkdc^scid^Il2rg^tm1Wjl^/SzJ,* NSG) mice were injected i.v. with 10^6^ U266-GFP-luc cells (U266 tumor group, *n* = 8) or PBS (NSG, naïve control group, *n* = 8). All mice were sacrificed at 10 wk posttumor cell injection. The U266 model and naïve mice (*n* = 8 or 3/group, respectively) were also used for quantitative real-time PCR (qPCR) of primary osteocytes.

All mice were randomized by weight and group-housed in individual cages with a 12-h light/dark cycle and had free access to food and water. Experimental mouse group numbers were calculated using power calculations based on previously published data.^22^ All analyses were performed blinded.

### μCT and high-resolution SR-μCT

Femora and tibiae were fixed in 10% formalin for 48 h and then stored in 70% ethanol. For ex vivo μCT, right femora were scanned on a Skyscan 1272 (Bruker, Switzerland) at 50 kV and 200 μA at 4.3-μm pixel resolution for a 180° scan with 0.7° rotation. A 1-mm region of interest (ROI) 0.4 μm from the growth plate was determined, and trabecular bone as a percentage of bone volume (BV/TV), trabecular thickness (Tb. Th), trabecular number (Tb. N), trabecular separation (Tb. Sp), cortical volume (Ct. V), and cortical thickness (Ct. Th) was assessed in this region as previously described.^23^ Bone lesion area as a proportion of total bone surface area (%) was assessed using Osteolytica software as previously detailed.^24^

Swiss Light Source (TOMCAT beamline, Paul Scherrer Institut, Switzerland, Proposal ID 20220399): Right 5TGM1 tibiae at the tibiofibular joint^25^^,^^26^ and right U266 femora at the metaphysis were scanned using SR-μCT in absorption and inline phase-contrast imaging mode with 0.65-μm pixel size to visualize both bone and osteocytes within lacunae.^27^^,^^28^ For each scan, 3000 projection images were captured over a 360° rotation with a fixed energy of 21 keV and an exposure time of 120 ms, at a sample-to-detector propagation distance of 40 mm.

Diamond Light Source (I13-2 beamline, Didcot, UK, Proposal MG31801): Right 5TGM1 femora were scanned at the metaphysis using SR-μCT with 1.625-μm pixel size. For each scan, 2000 projections were captured over a 360° rotation with a beam energy of 20 keV, a ring current of 300 mA, and an exposure time of 130 ms.

### SR-μCT image processing and analysis

SR-μCT datasets were processed, analyzed, and imaged using the software Dragonfly (v. 2022 for Windows, Object Research Systems Inc., Montreal, Canada).

To analyze the osteocyte lacunae, an ROI was selected for both the femoral metaphysis and the tibiofibular joint. For the femoral metaphysis, this was a 0.5-mm section (769 slices) 0.1 mm distally from the last visible remaining portion of the growth plate. For the tibiofibular joint, this was a 0.5-mm section (769 slices) 0.1 mm distally from where the fibula connected to the tibia. Femoral ROIs also went through the additional step of having their trabecular bone removed from the dataset, leaving only the cortex.

For both femoral metaphysis and tibiofibular joint ROI datasets, firstly, the cortex was segmented from the background by global thresholding so that a binary image remained, where cortex was white and background was black. A sweep was also performed so that only the largest object remained, removing any unwanted artifacts. Next, a mask of the bone was created, with all pores (not open to the outside) filled in. This was to serve as the base, so that when the original, porous, bone was subtracted from it only the lacunae would remain. Pores were then filtered by volume as reported by Hemmatian et al.^29^ with those of a volume less than 2000 μm^3^ classed as osteocyte lacunae. Those with a volume greater than 2000 μm^3^ were classed as intracortical canals and excluded. Pores with a volume less than 25 μm^3^ were assumed to be noise and were similarly excluded. Osteocyte lacunae were quantified for the following parameters: density (number per mm^3^ of cortical bone), proportion (% of cortical bone), mean volume (μm^3^), and sphericity (relative index, with 1 equaling a perfect sphere).

### Histomorphometry and immunohistochemistry

Left tibiae were decalcified, wax embedded, then 3-μm sections cut and stained for tartrate-resistant acid phosphatase (TRAP) and hematoxylin as previously described.^30^ Quantification of tumor burden, osteoclasts, and osteoblasts were assessed as previously described.^31^

For staining of the osteocyte LCN, 3-μm paraffin-embedded sections were dewaxed and exposed to Ploton silver staining.^32^ Sections were stained with 50% silver nitrate solution, and silver staining then developed in a 5% sodium thiosulphate solution. Sections were also counterstained with hematoxylin for visualization of osteocyte nuclei.

The LCN was analyzed using Osteomeasure (Osteometrics, Decatur, GA, USA), Fiji (v. 1.543 t, National Institutes of Health, USA),^33^ and Dragonfly. For quantification of LCN coverage, a 3-field 1.5-mm region, 1.5 mm from the growth plate, was assessed and microscope field images taken every 0.5 mm. Fiji was used to assess LCN area as a proportion of total bone area. Microscope field images were loaded and for each an ROI encapsulating the entirety of the bone area was drawn and any osteocytes and cement lines removed, so that only bone area and LCN remained. The image was then binarized with the LCN becoming white and the bone surface becoming black. LCN area as a proportion of total bone area was then calculated for each of the 3 fields, before the mean was calculated. For quantification of osteocyte canalicular length, canaliculi arising from each osteocyte lacuna and extending as a single, unbranched projection was traced using Osteomeasure. The mean length was determined by loading microscope field images and measuring all canaliculi from 3 osteocytes per field for a total of 9 osteocytes, before an average canaliculi length was calculated, as previously carried out by Dole et al.^8^ For 2D analysis of LCN orientation, microscope field images were loaded into Dragonfly and aligned so that osteocyte lacunae lay as straight to the horizontal plane as possible, with canaliculi thus perpendicular to them in the vertical plane. The image was segmented, so that the LCN became white and the surrounding bone became black, and 2D orientation analysis was then performed, with the angle of major axis of each canaliculus measured from the vertical in degrees. Proportion of canaliculi falling between −20° and 20° around the vertical out of all canaliculi was then assessed.

For sclerostin assessment, decalcified 3-μm paraffin-embedded tibial sections were dewaxed and treated with pepsin for antigen retrieval and 3% H_2_O_2_ for blocking of endogenous peroxidase activity, followed by 10% casein for nonspecific protein block. Sections were incubated with primary sclerostin antibody (1:500, R&D, AF1589) followed by incubation with a secondary biotinylated antibody (horse anti-goat IgG biotin, Vector BA – 100, 1:200). Sections were then treated with an avidin-conjugated peroxidase (VectaStain Elite ABC-HROP Kit, Vector Laboratories) and DAB (Vector, ImmPACT DAB, SK-4105). Sclerostin protein was stained in brown and sections were counterstained with hematoxylin. Slides were scanned on a NanoZoomer XR slide scanner (Hamamatsu, Shizuoka, Japan) at 40×, and sclerostin coverage and number of sclerostin-positive osteocytes^34^ were quantified using QuPath (v. 0.4.3).^35^ A 1.5-mm region, 250 μm from the growth plate, was assessed.

### Quantitative real-time PCR

Prior to sacrifice, presence of tumor and MBD was confirmed in U266 mice as previously described.^22^ Right femora (after removal of soft tissue, distal and proximal ends, and marrow) were snap frozen in liquid nitrogen and then homogenized (Precellys Evolution, Bertin Instruments, France) in tri reagent (guanidinium thiocyanate).^8^ RNA was extracted using phenol-chloroform and quantified using a Nanodrop spectrophotometer. cDNA was synthesized using a High-Capacity RNA-to-cDNA kit (ThermoFisher Scientific) and qPCR was performed on a QuantStudio 7 with Design and Analysis 2.6.0 software (Applied Biosystems, CA, USA) with TaqMan primers used for quantification of *Dmp1 (dentin matrix acidic phosphoprotein 1)*, *Sost*, *MMP13 (matrix metalloproteinase-13)*, *Ctsk (cathepsin K)*, *Acp5 (acid phosphatase 5, TRAP)*, and *MMP2 (matrix metalloproteinase-2)* genes relative to *Gapdh*.^36^

### Statistical analysis

All data are presented as mean ± SD. Statistical analysis was performed using GraphPad Prism 9 (GraphPad Software, Inc., La Jolla, CA, USA) using unpaired two-tailed Student’s *t*-test.

## Results

### Mice with 5TGM1 or U266 tumors exhibit MBD

**Figure 1:**
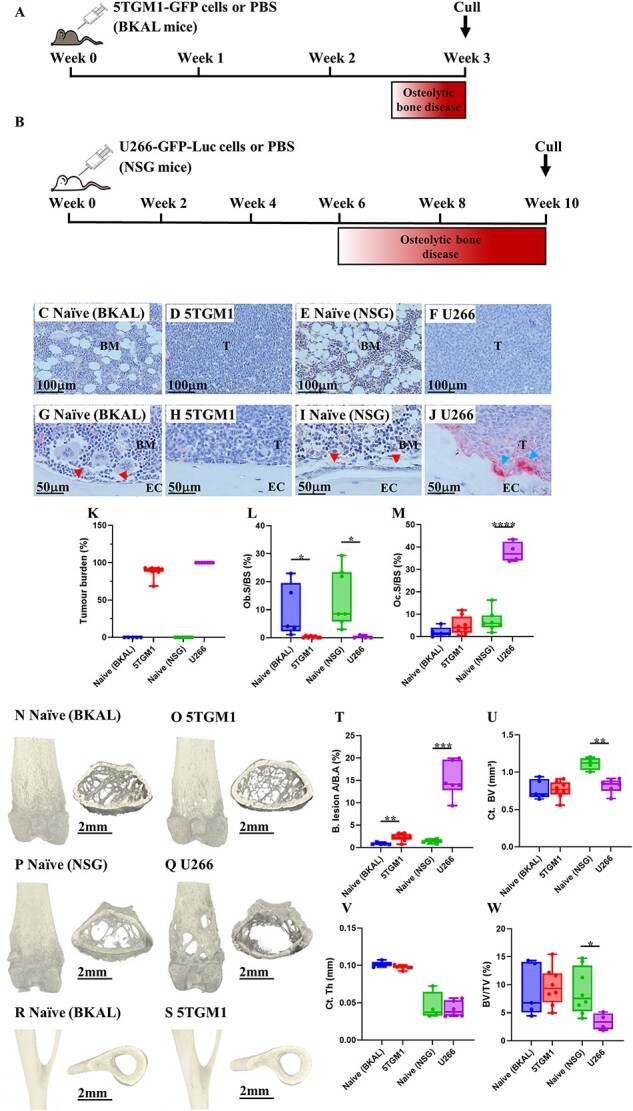
Extent of myeloma bone disease in 5TGM1 and U266 murine models. Study schematics of (A) 5TGM1 and (B) U266 models, BKAL and NSG mice were used as naïve control groups, respectively. Representative images of tibial hematoxylin-stained sections of bone marrow from (C) naïve (BKAL) and (D) 5TGM1, and (E) naïve (NSG) and (F) U266. Tibial TRAP-stained sections show endocortical bone surface of (G) naïve (BKAL) and (H) 5TGM1, and (I) naïve (NSG) and (J) U266, showing distribution of osteoblasts (arrows G, I) and osteoclasts (arrows J). Histomorphometric analysis of (K) tumor burden, (L) osteoblast surface, and (M) osteoclast surface. Representative images of bone lesions and trabecular structure found in the distal femur of (N) naïve (BKAL) and (O) 5TGM1, and (P) naïve (NSG) and (Q) U266; tibiofibular joint region of (R) naïve (BKAL) and (S) 5TGM1; and μCT analysis at the distal femur assessing (T) osteolytic lesion area, (U) cortical bone volume, (V) cortical thickness, and (W) trabecular bone volume. T = tumor, EC = endocortical. Data are presented as mean ± SD. ^*^p<.05, ^*^^*^p<.01, ^*^^*^^*^p<.001, ^*^^*^^*^^*^p<.0001 (unpaired two-tailed Student’s *t*-test).

To first verify that the 5TGM1 and U266 models (Figure 1A, B) successfully resulted in MBD, we assessed the bones for the presence of tumor cells, as well as changes in osteoblasts and osteoclasts (Figure 1C–M). Tumor burden was high in both 5TGM1 and U266 (Figure 1C–F, K), and both myeloma models exhibited decreased osteoblast surface coverage and osteoblast number (Figures 1G–J, L and S1A). Osteoclast surface coverage and number remained unchanged in the 5TGM1 model but rose significantly in the U266-bearing mice (Figures 1G–J, M and S1B). Both myeloma models developed osteolytic lesions in the proximal femur, although these were far more extensive (5-fold higher) in the U266 model (Figure 1N–Q, T). Neither model exhibited lesions in the tibiofibular joint region (Figure 1R, S). For cortical bone, 5TGM1-bearing mice showed no significant change in either cortical volume or cortical thickness compared with naïve, whereas U266-bearing mice exhibited a decreased cortical volume while cortical thickness remained unchanged when compared with naïve (Figure 1U, V). For trabecular bone architecture, 5TGM1-bearing mice showed no significant change in trabecular bone volume, but U266-bearing mice exhibited a lower trabecular bone volume (Figure 1W) than naïve controls. Trabecular thickness, trabecular number, and trabecular separation were unchanged between disease and naïve in both models (Figure S1C–E).

### Osteocyte lacunae are enlarged and more ellipsoidal in U266 mice

**Figure 2:**
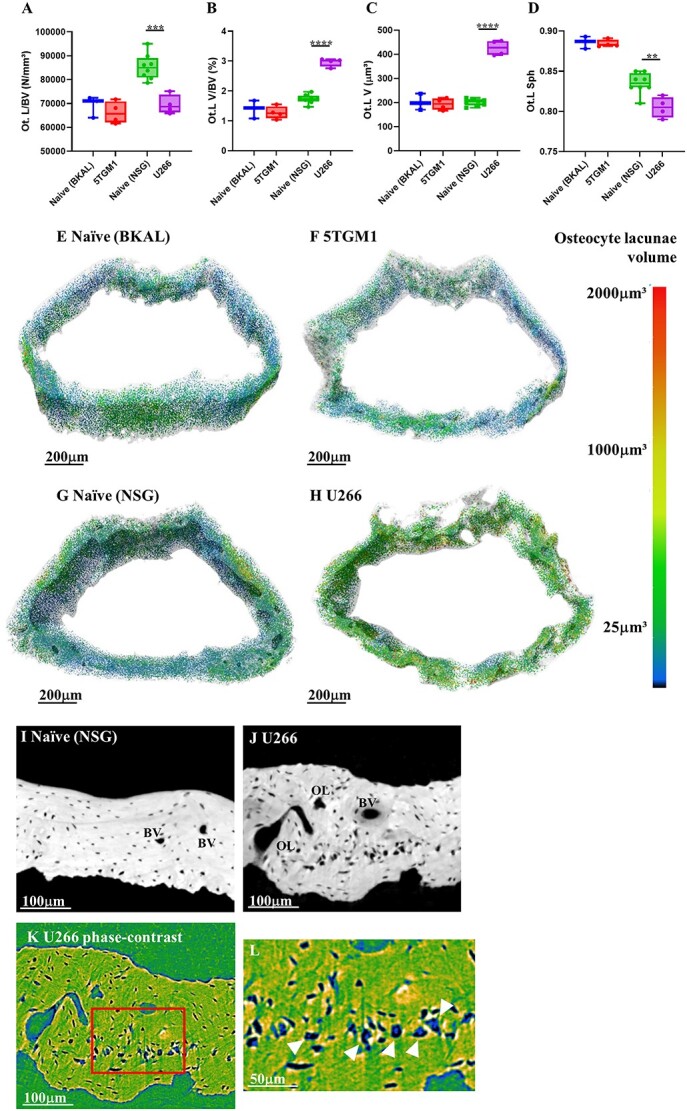
Osteocyte lacunae density is less in U266 mice with larger and more ellipsoidal lacunae. SR-μCT analysis of the osteocyte lacunae in 5TGM1, U266, or respective naive mice at the femoral metaphysis showing (A) osteocyte lacunae density, (B) osteocyte lacunae as a proportion of the bone, (C) average osteocyte lacunar volume, and (D) average osteocyte lacunar sphericity. Representative images of (E) naïve (BKAL), (F) 5TGM1, (G) naïve (NSG), and (H) U266 femoral metaphysis regions assessed by SR-μCT with individual osteocyte lacunae false color-mapped by volume. Representative SR-μCT images of (I) naïve (NSG) and (J) U266 mice femoral cortical bone showing typical osteocyte lacunae. Phase-contrast imaging of the same U226 bone using false-color mapping and (L) zoomed-in imaging showing osteocytes sitting within the enlarged osteocyte lacunae (arrows). BV = blood vessel, OL = osteolytic lesion. All data shown as mean ± SD. ^*^^*^p<.01, ^*^^*^^*^p<.001, ^*^^*^^*^^*^p<.0001 (unpaired two-tailed Student’s *t*-test).

To determine whether the presence of MBD induced changes in osteocyte lacunae, we examined the femoral metaphysis and tibiofibular joint by high-resolution SR-μCT scanning (Figure S1F–J). At the femoral metaphysis, osteocyte lacunar density was unaffected in 5TGM1 mice compared with naïve, but in U266 mice, it was significantly decreased (Figure 2A). Lacunar volume as a proportion of total bone volume was similarly unaffected in 5TGM1 compared with naïve but significantly increased in U266 (Figure 2B). This increase in proportion was driven by a significant doubling in average lacunar volume for U266 compared with naïve, while average lacunar volume remained unchanged for 5TGM1 (Figure 2C, E–H). Lacunar sphericity was also significantly altered in the U266 model with less spherical lacunae compared with naïve, while there was no change in sphericity in 5TGM1 (Figure 2D). We also examined the tibiofibular joint region in the 5TGM1 model compared with naïve (Figures 1R, S and S1G); this region typically does not develop MBD, and we wanted to test whether the changes in osteocytes found at the femoral metaphysis were systemic or localized. No changes in osteocyte lacunar density, proportion, volume, or sphericity were observed in this region (Figure S1K, P).

To confirm the enlarged lacunae in U266 bones still housed osteocytes and were not simply empty pits, the lacunae were examined in SR-μCT scans taken using phase-contrasting techniques, which allows the visualization of both high-contrast objects (bone) and low-contrast objects (osteocytes). Solid structures were clearly identifiable within the lacunae, which we believe to be osteocytes (Figure 2I, L).

### 5TGM1 and U266 mice have osteocyte canaliculi of a shorter appearance compared with naïve mice

**Figure 3:**
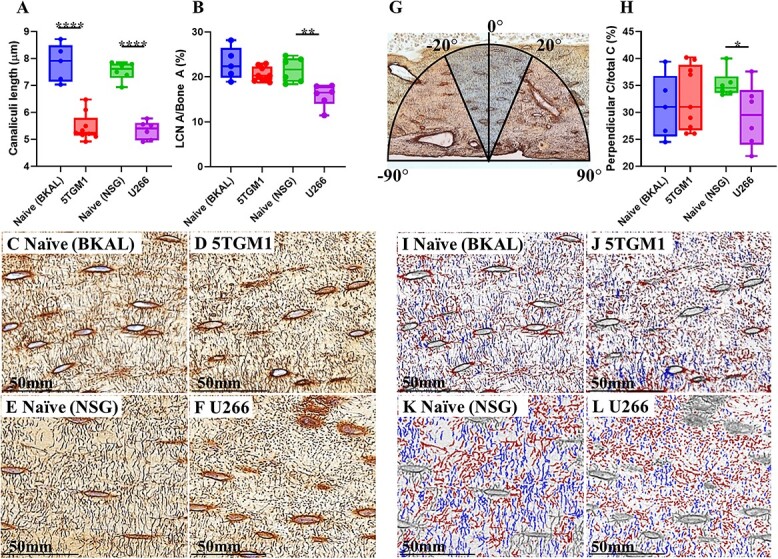
Myeloma bone disease leads to apparent shortening of osteocyte canalicular length and decreased LCN area coverage. Analysis of the LCN showing (A) canalicular length and (B) LCN area coverage. Representative Ploton silver nitrate-stained images of tibial cortical bone of (C) naïve (BKAL), (D) 5TGM1, (E) naïve (NSG), and (F) U266, showing osteocytes and the LCN. Analysis of LCN organization by (G) aligning osteocytes perpendicular to bone lamellae (0°) and assessing degrees of alignment of canaliculi to 0°, and (H) proportion of canaliculi aligned within 40° of 0°. Representative images of canalicular orientation of (I) naïve (BKAL), (J) 5TGM1, (K) naïve (NSG), and (L) U266, with aligned canaliculi in blue and unaligned canaliculi in red. All data shown as mean ± SD. ^*^p<.05, ^*^^*^p<.01, ^*^^*^^*^^*^p<.0001 (unpaired two-tailed Student’s *t*-test).

Osteocytes function as part of a complex dendritic network that allows them to communicate with each other. We assessed if MBD altered the coverage and structure of the LCN. We treated tibial sections with Ploton silver staining and examined a region at the tibial metaphysis. Average apparent canaliculi length was significantly shorter in both 5TGM1 (30.4%) and U266 (29.1%) tibiae compared with naïve tibiae (Figure 3A, C–F). LCN area as a proportion of the bone was also 24.2% lower in U266 tibiae compared with naïve, while 5TGM1 tibiae were unaffected (Figure 3B, C–F). To determine whether LCN orientation was altered, the number of canaliculi lying perpendicular to the osteocytes was measured as a proportion of all canaliculi (Figure 3G). U266 tibiae exhibited fewer perpendicular canaliculi compared with naïve, indicating altered LCN organization, while 5TGM1 LCN organization was unaffected (Figure 3H–L).

### Osteocytes from U266 femora have elevated expression of PLR genes

**Figure 4:**
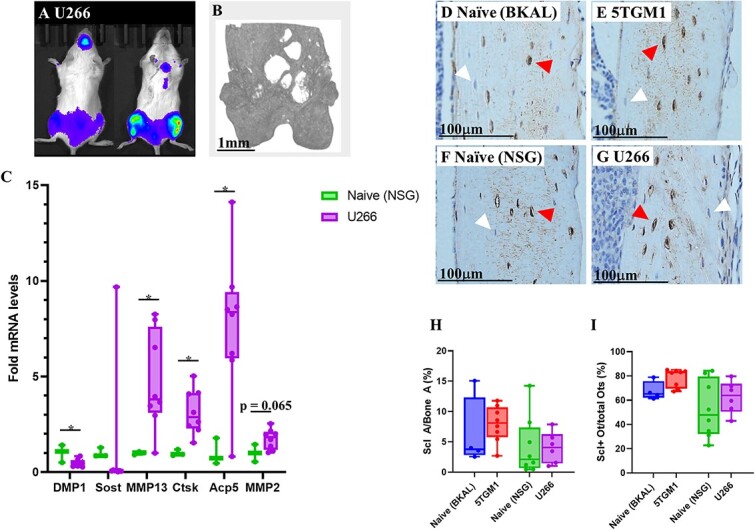
Myeloma bone disease leads to up-regulation of PLR-specific genes but not *Sost*. Representative bioluminescent imaging of U266 tumor burden (A) and in vivo μCT of the distal femur (B) showing bone lesions. (C) qPCR analysis of osteocyte-specific genes *Dmp1* and *Sost*, and PLR genes *MMP13*, *Ctsk*, *Acp5*, and *MMP2* relative to *Gapdh* expression. Representative images of sclerostin staining of tibial cortical bone of (D) naïve (BKAL), (E) 5TGM1, (F) naïve (NSG), and (G) U266, with red arrows indicating sclerostin-positive osteocytes and white arrows indicating sclerostin-negative osteocytes. Analysis of sclerostin IHC showing (H) proportional sclerostin staining coverage and (I) proportion of sclerostin-positive osteocytes. All data shown as mean ± SD. ^*^p<.05 (unpaired two-tailed Student’s *t*-test).

Osteocytes are known to contribute to bone homeostasis by PLR, a process whereby bone surrounding osteocytes is resorbed and deposited. Therefore, we investigated whether the increased lacunar size was associated with PLR. We isolated primary femoral osteocytes from the U266 and naïve (NSG) mice and performed qPCR (Figure 4A–C) for a panel of genes: *Dmp1* and *Sost*, which are osteocyte-specific; and *MMP13*, *Ctsk*, *Acp5* and *MMP2*, which are all genes known to be involved in PLR.^14^^,^^37–39^ It should be noted that the widely used primary osteocyte isolation technique^8^^,^^40^^,^^41^ does not exclude the possibility that other bone cells may be present in the population, such as osteoclasts. However, *Dmp1* and *Sost* were highly expressed in all samples, confirming a high purity of osteocytes in the population.^40^  *Dmp1* showed a 0.49-fold change in U266 compared with naïve. For the PLR genes, *MMP13* showed a 4.9-fold increase; *Ctsk* a 3.2-fold increase and *Acp5* a 7.7-fold increase compared with naïve. *MMP2*, although not significant, showed a trend for a 1.74-fold increase (*p*=.065). Despite the upregulation of PLR-specific genes, there was no significant change in *Sost* expression in U266 mice compared with naïve. We did not perform qPCR analysis on the primary osteocytes of 5TGM1 mice owing to their unchanged osteocyte lacunar morphology. To further examine the upregulation of *Acp5*, we examined histological sections of U266 bone after staining for TRAP and found numerous incidences of visible TRAP staining around the nuclei of osteocytes (Figure S1Q, R). This helps confirm that the osteocytes are performing osteocytic osteolysis and rules out the possibility that the larger osteocyte lacunae are due to newly formed osteocytes, which typically present with larger lacunae. However, it was impossible to quantify the TRAP staining around the osteocytes, owing to the TRAP staining being too faint as to be confidently identified in many areas.

### Increased osteocyte activity is not associated with increased sclerostin production

Since sclerostin is known to stimulate PLR,^14^ we next determined whether sclerostin protein levels were increased in U266 bones, though based on the expression data, we predicted that no significant differences would be seen between disease and naïve bones. Tibial metaphysis sections were stained for sclerostin by IHC (Figure 4D–G) and sclerostin area as a proportion of total bone area was found to be unchanged in disease tibiae (5TGM1 and U266) compared with respective naïves (Figure 4H). Osteocytes were also tagged as to whether they were positive or negative for sclerostin, and the proportion of sclerostin-positive osteocytes was found to be unaltered between disease and naïve tibiae (Figure 4I).

## Discussion

Bone disease affects over 90% of myeloma patients, exposing them to chronic pain and high risk of fracture. Current treatments for MBD are limited as they do not repair damaged bones, indicating an unmet clinical need. To improve the treatment of MBD, our fundamental knowledge of MBD, particularly the role of osteocytes, needs to improve. Here, we set out to understand more about the role of osteocytes and their LCN in MBD by comparing healthy bones to myeloma-bearing bones.

Initially, we determined the extent of MBD in two different mouse models and found that both models developed high levels of tumor burden but had differing levels of MBD: mild MBD in 5TGM1 and extensive MBD in U266. SR-μCT showed that osteocyte lacunae proximal to extensive MBD in U266 mice were greatly enlarged and more ellipsoid in comparison to naïve bones. This was not seen in 5TGM1. One reason for the discrepancy between models could be the extent of the osteolytic disease; however, Hemmatian et al.^42^ reported enlarged osteocyte lacunae in breast and prostate cancer models that both displayed extensive osteolytic disease, but the magnitude of the size change was much smaller than we have found in myeloma. Furthermore, they found that the larger lacunae were proximal to osteosclerotic regions rather than osteolytic regions. Another reason could be the timespan of the models used: U266 is a 9-wk model, whereas 5TGM1 and the breast and prostate models used by Hemmatian et al. are shorter 3-wk models, suggesting that the osteocyte lacunae require time to become fully enlarged. The discrepancy between the 5TGM1 and U266 model could be further tested by using the JJN3 model, which is a very aggressive 3-wk human xenograft model of myeloma that results in very severe MBD. Examining the osteocytes of JJN3 mice would help to confirm whether it is the severity of disease or length of disease course that are driving the changes. Finally, it cannot be discounted that 5TGM1 mice have a functional immune system, whereas U266 mice are immunocompromised, and that the presence of an immune system may be why fewer osteocyte and LCN changes are seen in the 5TGM1. This could be tested by examining osteocytes in a more long-course syngeneic model of myeloma such as the 5 T2 model^43^; however, access to this mouse model is very limited at present.

We found that substantial MBD decreased osteocyte lacunae density by ~20%, in keeping with what has been demonstrated clinically in patients.^15^ Vashishth et al. have shown that decreased osteocyte density is correlated to an increased accumulation of microcracks, indicating that a dense osteocyte network is linked to better bone quality.^44^ We then quantified, for the first time to our knowledge, that MBD leads to an apparent decreased osteocyte canalicular length and overall LCN coverage, and that extensive MBD causes the LCN to be organized differently; similar observational findings were described by Ziouti et al.^16^ The mechanisms behind this apparent canalicular shortening and disorganization are currently unknown, and more work is required to determine how it comes about. It should also be reiterated that the canaliculi were only assessed in 2D and that 3D analysis using a technique such as fluorochromes and confocal microscopy would be beneficial to confirm the shortened canaliculi. However, changes in the LCN such as reduced coverage and shortened canaliculi can affect the mechano-sensing perception of osteocytes, diminishing their ability to initiate appropriate bone remodeling in response to loading or microfracture.^45^ These findings may have further reaching clinical implications, as Ding et al. demonstrated in their partial osteocyte-ablation murine model that when osteocyte numbers were reduced, rapid development of osteoporosis and signs of early aging were observed.^46^

Osteocytes are known to help maintain bone homeostasis through processes such as sclerostin secretion and PLR. We found no changes either in *Sost* upregulation in osteocytes or localized sclerostin production, concurring with the findings of McDonald et al.^19^ but contradicting other reported findings. The role of sclerostin in myeloma is clearly complex, and these contrasting conclusions may be due to sclerostin levels fluctuating depending on the severity of MBD and the timepoint at which they are measured in the disease course. It is also true that sclerostin is expressed by other cells within the bone marrow, such as hypertrophic chondrocytes,^47^ and that MBD may further influence these expression pathways. However, we did determine that PLR-specific genes were upregulated in osteocyte-enriched populations from bones of extensive MBD-bearing mice. The resorption part-process of PLR (also known as osteocytic osteolysis) involves osteocytes secreting matrix metalloproteinases and enzymes such as cathepsin K and TRAP to dissolve the surrounding bone matrix, and the upregulation of these related genes provides evidence that PLR is contributing to bone loss in myeloma. To our knowledge, this is the first time that PLR has been proposed as a mechanism of MBD pathophysiology. Interestingly, since sclerostin is known to be a promotor of PLR and its levels in osteocytes were unaffected, other potential drivers of the observed increase in PLR are still to be identified. Aberrant PLR is clearly detrimental to bone health, and this leads to the question of whether PLR-specific genes could be targeted therapeutically in order to normalize osteocyte function. This opens up future avenues of exploration into the cause and consequence of PLR in myeloma.

In summary, we have identified that extensive MBD leads to a decrease in the osteocyte population and an enlargement of osteocyte lacunae. The osteocyte LCN is compromised by MBD, with decreased canalicular length, network area coverage, and altered organization. PLR-specific genes are upregulated in the osteocytes in MBD, and further work is needed to explore the ramifications of PLR in myeloma.

## Supplementary Material

Supplemental_Data_ziae093

## Data Availability

The authors confirm that the data supporting the findings of this study are available within the article [and/or] its supplementary materials.
