# Correction to: Evidence for peri-lacunar remodeling and altered osteocyte lacuno-canalicular network in mouse models of myeloma-induced bone disease

**DOI:** 10.1093/jbmrpl/ziae123

**Published:** 2024-09-24

This is a correction to: Holly Evans, Rebecca Andrews, Fatma Ali Abedi, Alexandria Sprules, Jacob Trend, Goran Lovric, Alanna Green, Andrew Chantry, Claire Clarkin, Janet Brown, Michelle Lawson, Evidence for peri-lacunar remodeling and altered osteocyte lacuno-canalicular network in mouse models of myeloma-induced bone disease, *JBMR Plus*, Volume 8, Issue 9, September 2024, https://doi.org/10.1093/jbmrpl/ziae093

In the originally published version of the manuscript, in the Author Contributions section, those for the first author were erroneously omitted.

The section has now been emended in the article to read additionally: ``Holly Evans (Investigation, Methodology, Formal analysis, Interpretation, Writing—original draft, Writing—review and editing). [...]''.

