# Supplementary material for: Evidence for peri-lacunar remodeling and altered osteocyte lacuno-canalicular network in mouse models of myeloma-induced bone disease
Source: JBMR Plus. 2024 Jul 12;8(9):ziae093. doi: 10.1093/jbmrpl/ziae093 (PMC11299509; doi:10.1093/jbmrpl/ziae093)
Supplement: Supplemental_Data_ziae093 [file supplemental_data_ziae093.docx]

**Supplementary figure legends**


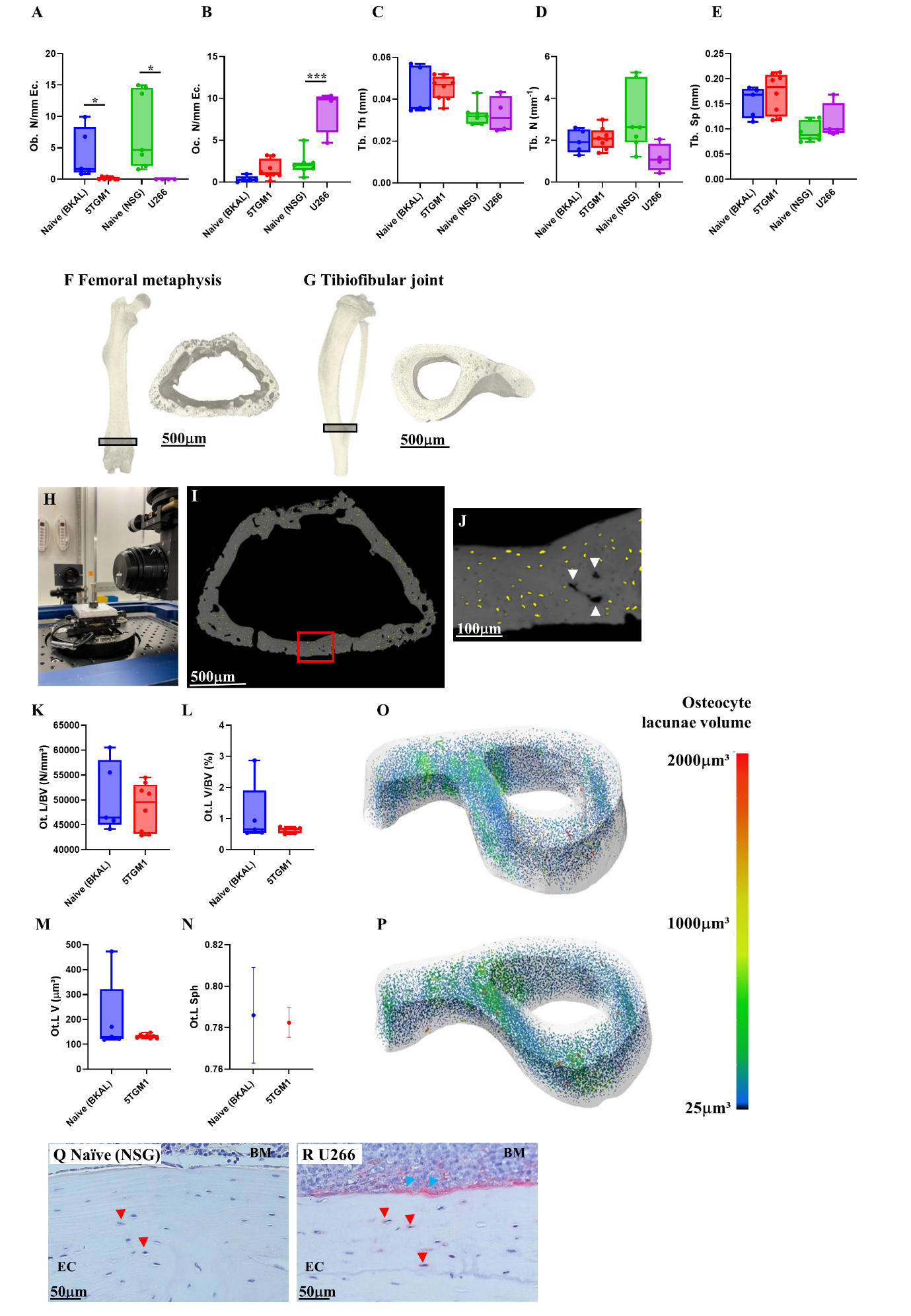


**Supplementary Figure 1. Osteocytes are unchanged at the TFJ in 5TGM1 mice.** Histomorphometric analysis of (A) osteoblast number and (B) osteoclast number. μCT analysis at the distal femur assessing (C) trabecular thickness, (D) trabecular number and (E) trabecular separation. Representative images of (F) the femoral metaphysis region scanned highlighted by the black box and the resulting 500μm region imaged and analyzed at 0.65μm resolution and (G) the tibiofibular joint region scanned highlighted by the black box and the resulting 500μm region imaged and analyzed at 0.65μm resolution. (H) Image of a femur to be scanned, mounted on a metal chuck and glued in place. Representative image of (I) the femoral metaphysis showing successfully isolated osteocyte lacunae in yellow and (J) a zoomed-in region showing the successful exclusion of blood vessels (white arrows). SR-μCT analysis of the osteocyte lacunae in 5TGM1 at the tibiofibular joint showing (K) osteocyte lacunae density, (L) osteocyte lacunae volume as proportion of the bone, (M) average osteocyte lacunar volume and (N) average osteocyte lacunar sphericity. Representative images of (O) naïve (BKAL) and (P) 5TGM1 tibiofibular joint regions with the individual osteocyte lacunae false color-mapped by volume. Representative images of tibial TRAP-stained sections of endocortical bone of (Q) naïve (NSG) and (R) U266, 1.5mm from the growth plate, showing osteocytes (red arrows) and TRAP staining (pink). Osteoclasts are also highlighted (blue arrows). BM=bone marrow, EC=endocortical. All data shown as mean ±SD. *<0.05, ***p<0.001 (unpaired two-tailed Student’s t-test).
